# Supplementary material for: Complexome profiling of the Chlamydomonas psb28 mutant reveals TEF5 as an early PSII assembly factor
Source: Plant Cell. 2025 Mar 17;37(6):koaf055. doi: 10.1093/plcell/koaf055 (PMC12164590; doi:10.1093/plcell/koaf055)
Supplement: koaf055_Supplementary_Data [file koaf055_supplementary_data.zip › psb28_interactive.zip!/plots/intensity/qualityPlots/peptides/COX90_Cre16.g691850.t1.2.html]

### Cre16.g691850.t1.2

Trivial name: COX90  
SeqCoverage [median]: 55.20  
Mass: 11.68 kDa  
  
 

| Sequence | MissedCleavage | Mass [Da] | wtIntensity | muIntensity | Score |
| --- | --- | --- | --- | --- | --- |
| EFLEEQAK | 0 | 992.48 | 51858.58 | 60786.49 | 191.63 |
| IFEVPLGYVVPR | 0 | 1387.79 | 39409.29 | 44412.55 | 176.33 |
| IPGSILGPEDAKPE | 1 | 1421.74 | 29531.47 | 71028.48 | 224.42 |
| MNAPPVYLNPFTNR | 0 | 1632.81 | 11033.02 | 11989.76 | 129.84 |
| MNAPPVYLNPFTNRIPGSILGPEDAKPE | 2 | 3036.54 | 2558.67 | 0.00 | 81.89 |
| WAAEHPTAPR | 0 | 1134.56 | 10341.25 | 18881.07 | 103.88 |
|  |  |  | 144732.3 | 207098.4 |  |
